# Clinical correlates for fluconazole resistance in *Candida* bloodstream infections

**DOI:** 10.3389/fpubh.2026.1963129

**Published:** 2026-10-05

**Authors:** Zihuan Li, Tian Wang, Mengdie Zhang, Shu Cao, Baohong Liu, Cuiqiong Fan, Guanwen Lin, Yanfen Ming

**Affiliations:** 1 Department of Infection Prevention and Control, The Affiliated Guangdong Second Provincial General Hospital of Jinan University, Guangzhou, Guangdong, China; 2 Outpatient Department, Longtan Community Health Service Station, Guangzhou, Guangdong, China; 3 Outpatient Department, South China University of Technology School of Medicine, Guangzhou, Guangdong, China; 4 Discipline Inspection Office, The Affiliated Guangdong Second Provincial General Hospital of Jinan University, Guangzhou, Guangdong, China

**Keywords:** bloodstream infection, *Candida*, fluconazole resistance, infection control, risk factors

## Abstract

**Background:**

Candidemia poses a growing global health threat, with rising fluconazole resistance complicating clinical management. This study aimed to investigate the species distribution and fluconazole resistance profiles of *Candida* bloodstream infections and identify risk factors associated with fluconazole resistance.

**Methods:**

A retrospective study was conducted at a tertiary care hospital in Guangzhou, China, from 2023 to 2025. Candida isolates from blood cultures were identified using MALDI-TOF MS, and antifungal susceptibility testing was performed using the VITEK 2 system. Patients were categorized into fluconazole-resistant and non-resistant groups based on species-specific MIC breakpoints.

**Results:**

A total of 94 *Candida* bloodstream infection cases were analyzed. The species distribution was: *Candida albicans* (26.60%), *Candida glabrata* (23.40%), *Candida auris* (20.21%), *Candida tropicalis* (15.96%), *Candida parapsilosis* (11.70%), and others (2.12%). Fluconazole resistance rates were highest in *Candida auris* (68.42%), followed by *Candida tropicalis* (46.67%) and *Candida parapsilosis* (9.09%). For *Candida auris* infections, indwelling medical devices (*p* = 0.017), antifungal use (*p* = 0.044), and antibacterial use (*p* = 0.004) were associated with fluconazole resistance. For *Candida tropicalis* infections, surgical history (*p* = 0.041) was the only factor associated with fluconazole resistance.

**Conclusion:**

*Candida auris* and *Candida tropicalis* demonstrated alarmingly high fluconazole resistance rates, while *Candida albicans* and *Candida glabrata* remained susceptible. Indwelling medical devices, prior antimicrobial exposure and antibacterial exposure were identified as factors associated with fluconazole resistance, particularly in *Candida auris* infections. These findings indicate that enhanced infection control measures are warranted.

## Introduction

*Candidemia* has emerged as a growing public health concern worldwide, imposing a substantial burden on healthcare systems (1). It is estimated that at least 600,000 cases of bloodstream infections caused by *Candida* species occur annually, with crude mortality rates reaching as high as 30–40% (2). Beyond the alarming mortality, these infections are also associated with prolonged hospitalization and substantially increased healthcare costs (3–8).

In recognition of the escalating threat posed by fungal infections, the World Health Organization (WHO) published its first Fungal Priority Pathogens List in 2022. This landmark document classified *Candida auris* (*C. auris*) and *Candida albicans* (*C. albicans*) as critical-priority pathogens, while *Candida tropicalis* (*C. tropicalis*) and *Candida glabrata* (*C. glabrata*) were designated as high-priority pathogens, underscoring the urgent need for enhanced surveillance, diagnostic capacity, and therapeutic strategies (2). National surveillance data from China between 2018 and 2021 further revealed considerable species-specific variability in antifungal resistance profiles. Notably, among isolates causing candidemia, the highest azole resistance rates were observed in *C. tropicalis*, with 42.5% resistant to fluconazole and 38.9% resistant to voriconazole (9).

Compounding these epidemiological concerns is the persistently limited antifungal armamentarium. Currently available antifungal agents, including amphotericin B, remain a cornerstone of therapy for severe invasive fungal infections. However, their clinical utility is frequently constrained by significant adverse effects (10). Against this backdrop, the progressive emergence of antifungal resistance, particularly among *Candida* species, and the recent nosocomial outbreaks of multidrug-resistant *C. auris* have collectively drawn unprecedented attention to this genus and elevated its priority status in both clinical and research agendas (2). Of particular concern is the increasing prevalence of fluconazole-resistant *Candida* strains, which have demonstrated the capacity to trigger hospital-based outbreaks accompanied by disproportionately high mortality rates (11, 12). Containing such outbreaks demands rigorous and resource-intensive infection control measures, further highlighting the critical need for robust antimicrobial stewardship programs, rapid diagnostic tools, and novel therapeutic agents to effectively manage this evolving threat.

There are limited data on Candida bloodstream infections in southern China. This study focuses on the species distribution and fluconazole resistance profiles of such infections in this region, aiming to provide an evidence-based reference for the rational selection of antifungal agents and the formulation of infection control strategies.

## Materials and methods

### Setting

This study was conducted at Guangdong Second Provincial General Hospital, a university-affiliated tertiary care hospital in Guangzhou, Guangdong Province, China, with approximately 1,730 beds. This retrospective study analyzed the number of *Candida* bloodstream infection cases and the prevalence of fluconazole resistance among these isolates during the period from 2023 to 2025.

#### Antimicrobial susceptibility testing

*Candida* isolates were identified to the species level using matrix-assisted laser desorption ionization–time of flight mass spectrometry (MALDI-TOF MS). Antifungal susceptibility testing was performed using the VITEK 2 antifungal susceptibility card, and the results were interpreted according to the Clinical Laboratory Standards Institute (CLSI) document, 3rd Edition, 2022. For *C. auris*, fluconazole MIC breakpoints were interpreted according to the CDC-recommended breakpoints (MIC ≥ 32 μg/mL). Quality control strains were tested in parallel, and all results fell within acceptable ranges.

Fluconazole resistance was defined based on species-specific minimum inhibitory concentration (MIC) breakpoints as follows: for *Candida albicans* (*C. albicans*), *C. parapsilosis*, and *C. tropicalis*, MIC ≥ 8 μg/mL; for *C. glabrata*, MIC ≥ 64 μg/mL; and for *C. auris*, MIC ≥ 32 μg/mL. In this study, isolates with MIC values at or above the respective breakpoints were classified as resistant, whereas those with MIC values below the breakpoints were classified as non-resistant.

#### Study population and inclusion criteria

Patients were enrolled if they met both of the following criteria: (1) a confirmed or suspected diagnosis of *Candida* bloodstream infection with a positive blood culture for *Candida* species, and (2) receipt of antifungal therapy. Exclusion criteria comprised (1) duplicate cases, (2) specimens considered likely contaminated, (3) absence of antifungal susceptibility testing results, and (4) age younger than 18 years. Based on fluconazole susceptibility, patients were categorized into a fluconazole-resistant group and a non-fluconazole-resistant group.

### Data collection

Clinical data of hospitalized patients with *Candida* isolates from blood cultures were extracted from the hospital-acquired infection intelligent management system (Beijing Ruilifang Medical Information Technology Co., Ltd.). All consecutive candidemia isolates recovered during the study period were included. The inclusion criterion of receipt of antifungal therapy was applied only to the analysis of antifungal exposure as a clinical correlate, not to the selection of isolates, so as to avoid selection bias toward more severe or resistant cases. Duplicate isolates from the same anatomical site and the same patient were excluded, and only the first isolate was retained for analysis. Baseline clinical data, including age, sex, length of hospital stay, and underlying risk factors, were obtained from the electronic medical record system V6.0 (Beijing Jiahui Meikang Information Technology Co., Ltd.). The evaluated risk factors comprised pre-existing comorbidities (hypertension, diabetes, renal insufficiency, hepatic insufficiency, etc.) and the presence of invasive indwelling devices (central venous catheters, urinary catheters, and endotracheal tubes) during hospitalization.

In addition, exposure to antimicrobial agents, including both antifungals and antibacterials, within the two-week period preceding a positive blood culture was recorded. All data were collected prior to the identification of *Candida* bloodstream infection.

### Statistical analyses

All statistical analyses were performed using IBM SPSS statistical software (version 29.0). Categorical variables were compared using the chi-square test. For categorical variables, the selection of statistical tests for 2 × 2 contingency tables followed standard criteria based on expected cell counts and total sample size: the Pearson chi-square test was applied when all expected cell counts were ≥5 and total sample size was ≥40; Yates’ continuity-corrected chi-square test was used when any expected cell count was between 1 and 5 (1 ≤ T < 5) and total sample size was ≥40; and Fisher’s exact test was employed when any expected cell count was <1 or total sample size was<40. Counted data were described by the number of cases (percentage), and the Kolmogorov–Smirnov test was used to verify the normality of the data. For continuous variables that followed a normal distribution, the mean ± standard deviation (SD) was used to describe them, and the differences between the groups were assessed using Student’s *t* test. For continuous variables that did not follow a normal distribution, data were described using the median and interquartile range (IQR, P25, P75), and group comparisons were performed using non-parametric tests, specifically the Mann–Whitney *U* test. A multivariable logistic regression analysis was conducted for *Candida*. Given the small sample size and the presence of complete separation in some comparisons, exact logistic regression was used for the multivariable analysis. All statistical analyses were evaluated at the statistical significance level of *p* < 0.05 (two-sided).

## Results

### Species distribution and fluconazole resistance profiles of *Candida* bloodstream infections

The species composition of *Candida* bloodstream infections is summarized as follows: *C. albicans* (*n* = 25, 26.60%), *C. glabrata* (*n* = 23, 23.40%), *C. auris* (*n* = 19, 20.21%), *C. tropicalis* (*n* = 15, 15.96%), and *C. parapsilosis* (*n* = 11, 11.70%), with *Candida krusei* (*C. krusei*) and *Candida famata* (*C. famata*) each accounting for one isolate (1.06%) (Figure 1A). With respect to fluconazole susceptibility, all *C. albicans* and *C. glabrata* isolates were classified as non-resistant. Among the other species, fluconazole resistance was detected in 13 of 19 *C. auris* isolates (68.42%), 7 of 15 *C. tropicalis* isolates (46.67%), and 1 of 11 *C. parapsilosis* isolates (9.09%) (Figure 1B). Figure 2 illustrates the fluconazole resistance profiles and MIC distributions for each *Candida* species.

**Figure 1:**
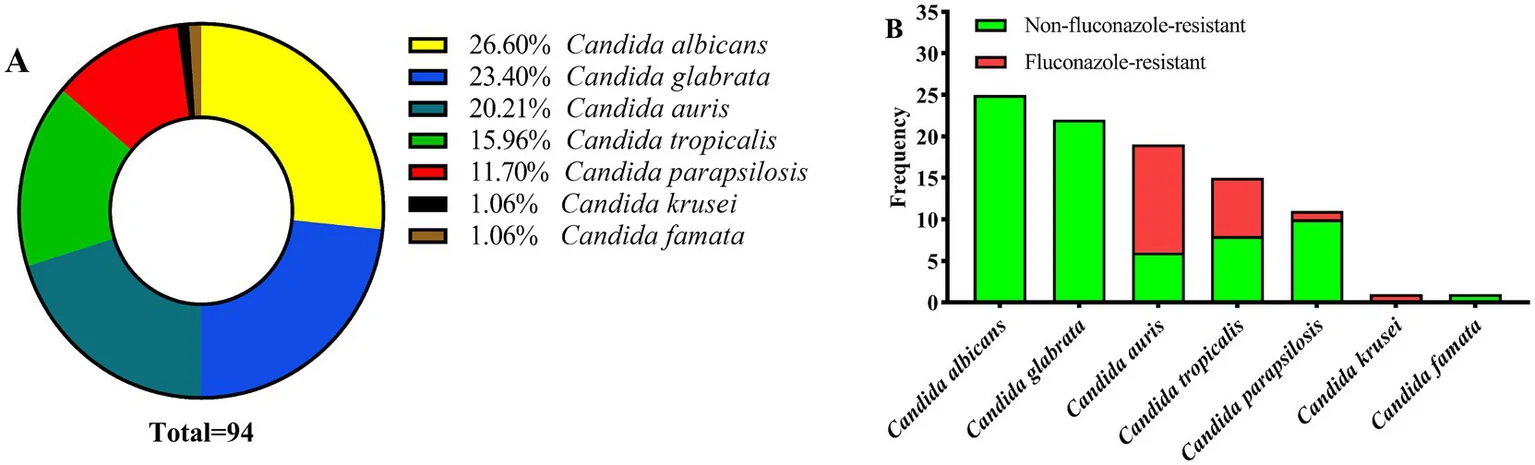
**(A)** Species distribution and **(B)** fluconazole resistance profiles of *Candida* bloodstream infections.

**Figure 2:**
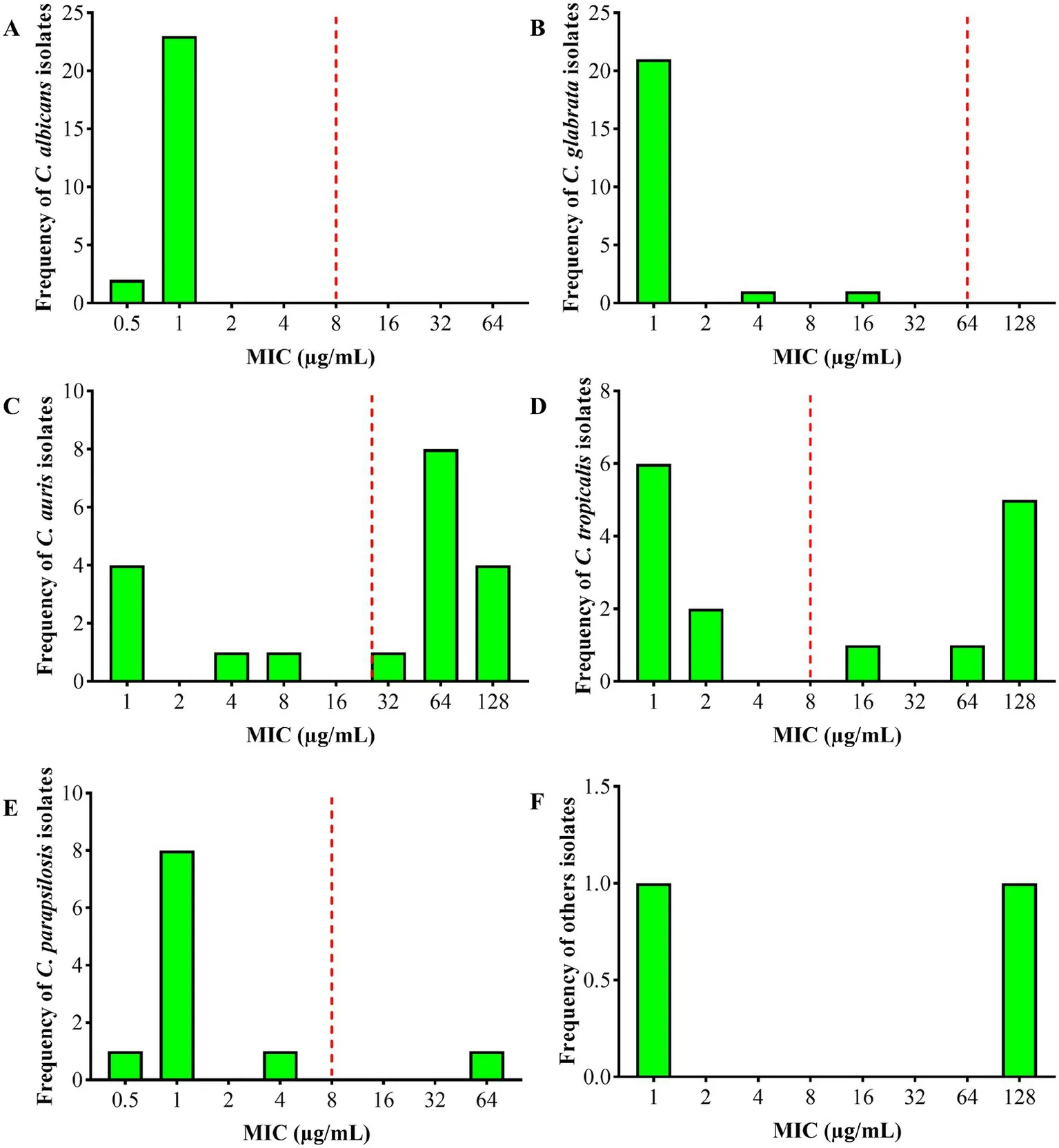
Distribution of MIC values for fluconazole. **(A)** *Candida albicans*. **(B)** *Candida glabrata*. **(C)** *Candida auris*. **(D)** *Candida tropicalis*. **(E)** *Candida parapsilosis*. **(F)** *Candida krusei* and *Candida famata*.

#### Baseline clinical characteristics

Comparisons of clinical characteristics between patients with *C. albicans* and non-*C. albicans* bloodstream infections are detailed in Table 1. Most clinical correlates were comparable between the two groups, except for *hepatitis*, which showed a statistically significant difference (*p* = 0.009). However, this finding should be regarded as hypothesis-generating given the number of comparisons performed. None of these variables remained statistically significant in the multivariable analysis (*p* > 0.05).

**Table 1:** Comparison of clinical characteristics between *C. albicans* and non-*C. albicans* bloodstream infection groups.

Characteristics	*Candida albicans* group (*n* = 25)	Non-albicans Candida group (*n* = 69)	*p* value
Age [mean ± SD]	70.00 [54.00, 82.00]	69.00 [56.00, 79.00]	0.853
Sex, Male—*n* (%)	17 (68.00)	44 (63.77)	0.704
Length of stay, days, median [IQR]	14.21 [8.46, 25.52]	15.50 [7.60, 24.39]	0.747
Pre-existing comorbidities—no. (%)			
Diabetes mellitus	7 (28.00)	21 (30.43)	0.820
Hypertension	11 (44.00)	28 (40.58)	0.766
Renal disease	4 (16.00)	12 (17.39)	1.000
Chronic liver disease	2 (8.00)	4 (5.80)	1.000
Tumor	5 (20.00)	17 (24.64)	0.639
Surgical history	14 (56.00)	36 (52.17)	0.743
Drug allergy history	2 (8.00)	3 (4.35)	0.859
Transfusion history	3 (12.00)	13 (18.84)	0.639
Hepatitis	7 (28.00)	4 (5.80)	0.009
Invasive procedures—no. (%)			
Indwelling medical devices	17 (68.00)	43 (62.32)	0.613
Antimicrobial use			
Antifungal use	7 (28.00)	21 (30.43)	0.820
Antibacterial use	21 (84.00)	50 (72.46)	0.250

Table 2 summarizes the comparison of clinical characteristics between patients with non-fluconazole-resistant and fluconazole-resistant *C. auris* bloodstream infections. Indwelling medical devices (*p* = 0.017), antifungal use (*p* = 0.044), and antibacterial use (*p* = 0.004) were all significantly associated with fluconazole resistance. None of these variables remained statistically significant in the multivariable analysis (all *p* > 0.05). As shown in Figure 3, the annual number of *C. auris* bloodstream isolates increased from 2 in 2023 to 7 in 2024 and 10 in 2025. In contrast, the proportion of fluconazole-resistant isolates decreased over the same period, from 100% (2/2) in 2023 to 85.71% (6/7) in 2024 and 50.00% (5/10) in 2025.

**Table 2:** Comparison of clinical characteristics between non-fluconazole-resistant and fluconazole-resistant *C. auris* bloodstream infection groups.

Characteristics	Non-fluconazole-resistant group (*n* = 6)	Fluconazole-resistant group (*n* = 13)	*p* value
Age [mean ± SD]	64.83 ± 21.74	74.08 ± 15.35	0.299
Sex, Male—*n* (%)	4 (66.67)	9 (69.23)	1.000
Length of stay, days, median [IQR]	23.93 [12.73, 36.96]	15.69 [12.78, 27.87]	0.416
Pre-existing comorbidities—no. (%)			
Diabetes mellitus	0 (0.00)	1 (7.69)	1.000
Hypertension	3 (50.00)	8 (61.54)	1.000
Renal disease	2 (33.33)	4 (30.77)	1.000
Chronic liver disease	1 (16.67)	2 (15.38)	1.000
Tumor	1 (16.67)	2 (15.38)	1.000
Surgical history	4 (66.67)	6 (46.15)	0.628
Drug allergy history	1 (16.67)	1 (7.69)	1.000
Transfusion history	3 (50.00)	3 (23.08)	0.320
Hepatitis	2 (33.33)	1 (7.69)	0.222
Invasive procedures—no. (%)			
Indwelling medical devices	2 (33.33)	12 (92.31)	0.017
Antimicrobial use			
Antifungal use	0 (0.00)	7 (53.85)	0.044
Antibacterial use	2 (33.33)	13 (100)	0.004

**Figure 3:**
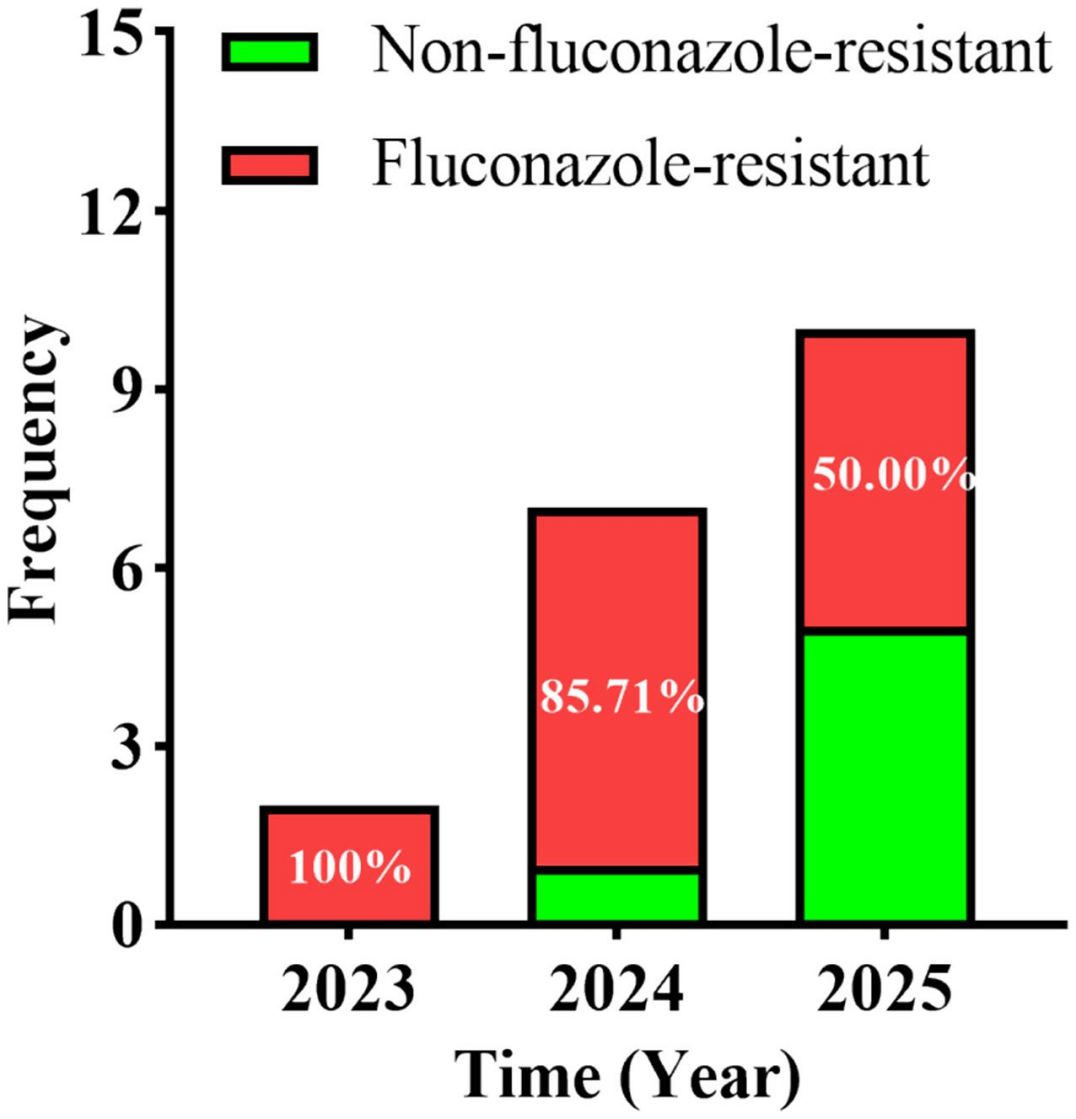
Temporal trends in non-fluconazole-resistant and fluconazole-resistant *C. auris* bloodstream infection groups.

Table 3 displays the comparison of clinical characteristics between patients with non-fluconazole-resistant and fluconazole-resistant *C. tropicalis* bloodstream infections. Surgical history (*p* = 0.041) was the only clinical correlate.

**Table 3:** Comparison of clinical characteristics between non-fluconazole-resistant and fluconazole-resistant *C. tropicalis* bloodstream infection groups.

Characteristics	Non-fluconazole-resistant group (*n* = 8)	Fluconazole-resistant group (*n* = 7)	*p* value
Age [mean ± SD]	63.63 ± 17.16	66.86 ± 19.79	0.740
Sex, Male—*n* (%)	4 (50.00)	2 (28.57)	0.608
Length of stay, days, median [IQR]	16.87 ± 10.22	33.73 ± 31.71	0.177
Pre-existing comorbidities—no. (%)			
Diabetes mellitus	5 (62.50)	4 (57.14)	1.000
Hypertension	3 (37.50)	2 (28.57)	1.000
Renal disease	2 (25.00)	0 (0.00)	0.467
Chronic liver disease	0 (0.00)	1 (14.29)	0.467
Tumor	3 (37.50)	1 (14.29)	0.569
Surgical history	6 (75.00)	1 (14.29)	0.041
Drug allergy history	0 (0.00)	0 (0.00)	-
Transfusion history	2 (25.00)	1 (14.29)	1.000
Hepatitis	1 (12.50)	0 (0.00)	1.000
Invasive procedures—no. (%)			
Indwelling medical devices	4 (50.00)	7 (100)	0.077
Antimicrobial use			
Antifungal use	3 (37.50)	4 (57.14)	0.619
Antibacterial use	7 (87.50)	6 (85.71)	1.000

## Discussion

Our study provides important insights into the species distribution and fluconazole resistance profiles of *Candid*a bloodstream infections in a tertiary care hospital in southern China. Among 94 candidemia cases, *C. albicans* (26.60%) remained the most prevalent species, followed by *C. glabrata* (23.40%), *C. auris* (20.21%), *C. tropicalis* (15.96%), and *C. parapsilosis* (11.70%). Alarmingly, fluconazole resistance rates were high in *C. auris* (68.42%) and *C. tropicalis* (46.67%), whereas all *C. albicans* and *C. glabrata* isolates remained susceptible. Indwelling medical devices, prior antifungal use, and antibacterial exposure were identified as factors associated with fluconazole resistance, particularly in *C. auris* infections. These findings carry important implications for empiric antifungal therapy selection and infection control practices.

Consistent with our findings, *C. albicans* remains the most frequently isolated species, while the proportion of non-albicans *Candida* species has been increasing (13–15). Geographically, *C. albicans* predominates in the Americas, Europe, and Africa, whereas *C. tropicalis* is more commonly isolated in Southeast Asia (16). Although *C. albicans* generally maintains susceptibility to antifungal therapy, non-albicans *Candida* species tend to exhibit reduced susceptibility, particularly to azoles (17).

The emergence of resistance in *C. parapsilosis* represents another serious concern. In our study, the fluconazole resistance rate for *C. parapsilosis* was 9.09%. Notably, one investigation reported a 36.84% increase in the incidence of *C. parapsilosis* infections (13). Prior to 2019, only sporadic outbreaks of fluconazole-resistant *C. parapsilosis* had been documented, yet these reports underscore the urgent need for ongoing resistance surveillance (17, 18).

On a global scale, *C. tropicalis* has demonstrated prominent azole resistance, with fluconazole non-susceptibility rates ranging from 30 to 35% in published studies (19, 20). In our cohort, however, the resistance rate of *C. tropicalis* to fluconazole reached 46.67%, which is somewhat higher than previously reported figures (3, 13). Given that azole antifungals remain the mainstay of first-line empirical therapy, the increasing azole resistance among *Candida* species not only compromises the efficacy of initial treatment but also substantially elevates the risk of therapeutic failure.

Regarding *C. auris*, our study observed a fluconazole resistance rate of 68.42%, reflecting a markedly high level of azole non-susceptibility. This is consistent with a report from South Africa, where the proportion of *C. auris* bloodstream infections increased significantly from 17% in 2019 to 31% in 2021 (1, 27). *C. auris* is recognized as a formidable pathogen due to its ability to contaminate the hospital environment, form biofilms on medical devices, colonize healthcare workers’ hands, and facilitate healthcare-associated outbreaks (21, 22).

In our study, indwelling medical devices, prior antifungal use, and antibacterial exposure were identified as significant risk factors for fluconazole resistance, particularly in *C. auris* infections. These findings are consistent with previously published reports (23–25). Additionally, the median age of patients in the *C. albicans* group was 70.00 years (IQR: 54.00–82.00), while that of the non-albicans *Candida* group was 69.00 years (IQR: 56.00–79.00). In line with other investigations, our study also observed a notably high prevalence of *Candida* bloodstream infections among older adult patients, further underscoring the heightened vulnerability of this population to invasive candidiasis (26).

The present study has several limitations that should be acknowledged. First, the retrospective, single-center design limits the generalizability of our findings to other populations and healthcare settings. Second, the relatively small sample size, particularly for subgroup analyses of *C. tropicalis* and *C. auris*, may have reduced statistical power to detect additional clinical correlates. Third, the absence of molecular characterization, such as genomic sequencing, precludes definitive identification of resistance mechanisms. Fourth, mortality and other clinical outcome data were not collected in this study; therefore, we were unable to assess the impact of fluconazole resistance on patient outcomes, particularly mortality. Given that the clinical significance of resistance is largely mortality-driven, this is a major limitation. The associations identified in this study should therefore be regarded as exploratory microbiological findings rather than conclusions regarding clinical outcomes. Fifth, our study did not capture detailed data on antifungal dosing, duration of therapy, or therapeutic drug monitoring, all of which may influence treatment outcomes.

## Conclusion

*Candida auris* and *Candida tropicalis* demonstrated alarmingly high fluconazole resistance rates, while *Candida albicans* and *Candida glabrata* remained susceptible. Indwelling medical devices, prior antimicrobial exposure and antibacterial use were identified as clinical correlates for fluconazole resistance, particularly in *Candida auris* infections. These findings indicate that enhanced infection control measures are warranted.

## Data Availability

The original contributions presented in the study are included in the article/supplementary material, further inquiries can be directed to the corresponding author.
